# External Male Genitalia in Henoch–Schönlein Syndrome: A Systematic Review

**DOI:** 10.3390/children9081154

**Published:** 2022-07-30

**Authors:** Valentina M. L. Montorfani-Janett, Gabriele E. Montorfani, Camilla Lavagno, Gianluca Gualco, Mario G. Bianchetti, Gregorio P. Milani, Sebastiano A. G. Lava, Marirosa Cristallo Lacalamita

**Affiliations:** 1 Family Medicine, Faculty of Biomedical Sciences, Università della Svizzera Italiana, 6900 Lugano, Switzerland; valentinajanett@hotmail.com (V.M.L.M.-J.); gabrielemontorfani@gmail.com (G.E.M.); mario.bianchetti@usi.ch (M.G.B.); 2 Pediatric Emergency Department, University Children’s Hospital Zurich, 8032 Zurich, Switzerland; camilla.lavagno@gmail.com; 3 Pediatric Institute of Southern Switzerland, Ente Ospedaliero Cantonale, 6500 Bellinzona, Switzerland; gianluca.gualco@eoc.ch; 4 Faculty of Biomedical Sciences, Università della Svizzera Italiana, 6900 Lugano, Switzerland; 5 Pediatric Unit, Fondazione IRCCS Ca’ Granda Ospedale Maggiore Policlinico, 20122 Milan, Italy; milani.gregoriop@gmail.com; 6 Department of Clinical Sciences and Community Health, Università degli Studi di Milano, 20122 Milan, Italy; 7 Pediatric Cardiology Unit, Department of Pediatrics, Centre Hospitalier Universitaire Vaudois and University of Lausanne, 1011 Lausanne, Switzerland; 8 Heart Failure and Transplantation, Department of Paediatric Cardiology, Great Ormond Street Hospital, London WC1N 3JH, UK; 9 Imaging Institute of Southern Switzerland, Ente Ospedaliero Cantonale, 6500 Bellinzona, Switzerland; marirosa.cristallolacalamita@eoc.ch

**Keywords:** Henoch–Schönlein syndrome, immunoglobulin a purpura, vasculitis, scrotum, penis, external genitalia

## Abstract

The external genitalia are notoriously implicated in every fifth male with Henoch–Schönlein syndrome. Nonetheless, the underlying conditions are poorly categorized. To characterize the involvement of the external male genitalia in this vasculitis, we performed a systematic review of the literature. For the final analysis, we selected 85 reports published between 1972 and 2022, which reported on 114 Henoch–Schönlein cases (≤ 18 years, N = 104) with a penile (N = 18), a scrotal (N = 77), or both a penile and a scrotal (N = 19) involvement. The genital involvement mostly appeared concurrently with or after the cutaneous features of Henoch–Schönlein syndrome, while it preceded the presentation of Henoch–Schönlein syndrome in 10 cases. Patients with penile involvement (N = 37) presented with swelling (N = 26), erythema (N = 23), and purpuric rash (N = 15). Most patients were otherwise asymptomatic except for transient micturition disorders (N = 2) or priapism (N = 2). Patients with scrotal involvement (N = 96) presented with pain (N = 85), swelling (N = 79), erythema (N = 42), or scrotal purpura (N = 22). The following scrotal structures were often involved: scrotal skin (N = 83), epididymis (N = 49), and testes (N = 39). An ischemic testicular damage was noted in nine patients (four with torsion and five without). The scrotal skin involvement was mostly bilateral, while that of the epididymis and testis were mostly (*p* < 0.0001) unilateral (with a significant predilection for the left side). In conclusion, this analysis allows for better categorization of the involvement of external male genitalia in Henoch–Schönlein vasculitis. Scrotal involvement can result from skin inflammation, epididymitis, orchitis, or testicular ischemia.

## 1. Introduction

Henoch–Schönlein syndrome, nowadays known also as immunoglobulin A purpura, is the most common form of small-vessel leukocytoclastic vasculitis in childhood. The hallmarks of the disease include palpable purpura concentrated in dependent areas, abdominal pain, joint pain or swelling, and glomerular kidney disease [1,2].

Allen is generally credited as the first to recognize, in 1960, the involvement of male external genitalia in Henoch–Schönlein syndrome [3]. However, two cases were reported before First World War [4,5]. A scrotal or penile swelling with erythema or pain occurs in approximately 20% of males with Henoch–Schönlein syndrome and is usually self-limited [1,2]. Yet, the underlying conditions are only poorly defined. To characterize the involvement of the external male genitalia in this form of vasculitis, we performed a systematic review of the literature.

## 2. Materials and Methods

### 2.1. Data Source

To increase the rigor of the work, we undertook this review in agreement with the 2020 version of the Preferred Reporting Items for Systematic Reviews and Meta-Analyses methodology, a set of items for reporting in systematic reviews and meta-analyses [6]. Three databases, namely Excerpta Medica, the United States National Library of Medicine, and the Web of Science were searched without limitations for original articles or letters published after 1960 using the following terms entered in separate pairs: (“anaphylactoid purpura” OR “Henoch–Schönlein” OR “Henoch” OR “IgA purpura” OR “IgA vasculitis” OR “immunoglobulin A purpura” OR “immunoglobulin A vasculitis” OR “rheumatoid purpura” OR “Schönlein–Henoch”) AND (“epididymis” OR “external genitalia” OR “penile” OR “penis” OR “penoscrotal” OR “scrotal” OR “scrotum” OR “testicles”). References listed within bibliographies of the retrieved records and relevant articles on authors’ personal files were also considered for inclusion. Furthermore, to detect as many cases as possible relating to this form of vasculitis, articles that were published in journals but not indexed in databases were also evaluated [7].

### 2.2. Eligibility Criteria and Data Extraction

Eligible sources were defined as original articles or letters reporting previously healthy males with the distinctive features of Henoch–Schönlein syndrome associated with a properly documented involvement of the external genitalia, i.e., scrotal skin, scrotal content, or penis.

The diagnosis of Henoch–Schönlein syndrome [1,2] made in the original reports was reviewed using recognized criteria (palpable purpura along with at least one of the following symptoms: abdominal pain, acute arthritis or arthralgia in any joint, or kidney involvement as evidenced by pathological hematuria with or without pathological proteinuria). A biopsy study was not a prerequisite for the diagnosis.

From each reported case of Henoch–Schönlein syndrome with involvement of the external genitalia, a prospectively defined schedule was used to excerpt data on age; pre-existing chronic conditions; infections (usually an upper respiratory infection, vaccinations or hymenoptera stings preceding the disease by ≤14 days); cutaneous, abdominal, articular, kidney, penile or scrotal involvement; imaging studies; intraoperative findings; histopathology and immunofluorescence findings; management; and course. The **C**utaneous, **A**bdominal, **A**rticular and **R**enal involvements [1] were scored on the **CAAR** grading scale (Table 1). The time interval between appearance of skin lesions and genital involvement was recorded. The clinical data, i.e., medical history, symptoms and signs (such as swelling, erythema, pain, difficult micturition or priapism), the intraoperative findings, and the results of imaging studies were used to categorize the involvement of penis, scrotal skin, and scrotal content, i.e., epididymis, spermatic cord, and testes [8,9]. The existence of an associated hydrocele was also addressed.

Two authors separately performed the literature search, the selection of articles retained for the analysis, and the data extraction. Any discrepancies were resolved through discussion until consensus was reached. If needed, a senior author was consulted. One author entered the data into a spreadsheet and the second author verified the accuracy of data entry.

### 2.3. Completeness of Reporting—Analysis

According to our standard procedure, the comprehensiveness in reporting each case was graded as satisfactory, good, or high [10].

Categorical variables are reported as proportions and continuous variables as medians with interquartile ranges. Dichotomous categorical variables were compared using the Fisher exact test; ordered categorical variables were compared using the Kruskal–Wallis test and the post hoc Bonferroni–Dunn correction [11]. A two-sided significance level of 0.05 was used.

## 3. Results

### 3.1. Search Results

**Figure 1:**
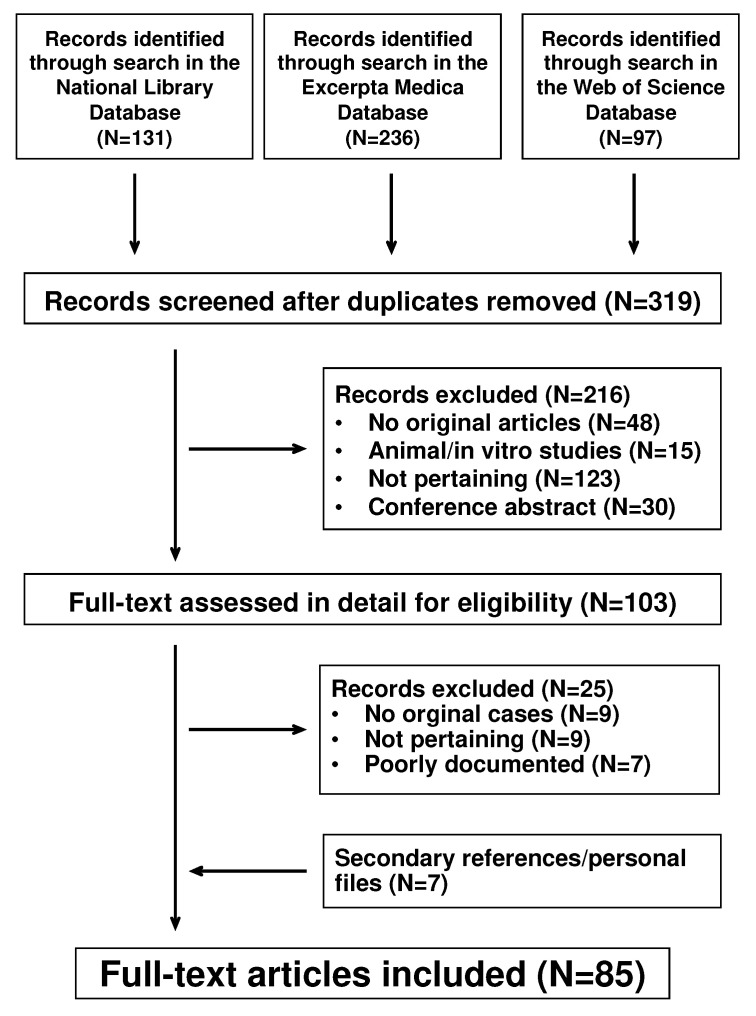
Involvement of external genitalia in males with Henoch–Schönlein syndrome. Flowchart of the literature search process.

The literature search returned 319 potentially relevant records (Figure 1). For the final analysis, we selected 85 reports published between 1972 and 2022 [12,13,14,15,16,17,18,19,20,21,22,23,24,25,26,27,28,29,30,31,32,33,34,35,36,37,38,39,40,41,42,43,44,45,46,47,48,49,50,51,52,53,54,55,56,57,58,59,60,61,62,63,64,65,66,67,68,69,70,71,72,73,74,75,76,77,78,79,80,81,82,83,84,85,86,87,88,89,90,91,92,93,94,95,96]: 44 from Europe (Turkey, N = 13; Spain, N = 9; Italy, N = 7; United Kingdom, N = 5; Switzerland, N = 3; Austria, N = 2; Ireland, N = 1; The Netherlands, N = 1; Norway, N = 1; Portugal, N = 1; Wales, N = 1), 21 from America (United States of America, N = 18; Canada, N = 3), 18 from Asia (Japan, N = 8; Israel, N = 3; South Korea, N = 2; India, N = 1; Islamic Republic of Iran, N = 1; People’s Republic of China, N = 1; Republic of China, N = 1; Saudi Arabia, N = 1), and 2 from Oceania (Australia, N = 2). They were published in English (N = 66), Spanish (N = 9), Turkish (N = 4), Italian (N = 3), French (N = 1), German (N = 1), and Norwegian (N = 1). A total of 114 male Henoch–Schönlein patients with involvement of the external genitalia were reported in the 85 articles. Reporting completeness was graded as satisfactory in 20 (17%), good in 58 (51%), and high in the remaining 36 (32%) cases.

### 3.2. General Data

#### 3.2.1. General Data

The characteristics of the 114 patients, composed of 104 (91%) children and 10 (9%) adults, appear in Table 2. An isolated penile involvement was observed in 18 [12,13,14,15,16,17,18,19,20,21,22,23,24,25], an isolated scrotal involvement in 77 [26,27,28,29,30,31,32,33,34,35,36,37,38,39,40,41,42,43,44,45,46,47,48,49,50,51,52,53,54,55,56,57,58,59,60,61,62,63,64,65,66,67,68,69,70,71,72,73,74,75,76,77,78,79,80,81,82,83], and a penoscrotal involvement in the remaining 19 [84,85,86,87,88,89,90,91,92,93,94,95,96] cases. An infection or, more rarely, a vaccine or a hymenoptera sting, preceded the clinical onset of the vasculitis in approximately 40% of cases. Genital involvement preceded the characteristic presentation of Henoch–Schönlein syndrome in no more than 10% of cases. Cutaneous, articular, abdominal, and kidney involvement were mostly mild to moderate.

Subjects with scrotal, penile, or penoscrotal involvement did not significantly differ with respect to age, precursors, and severity of cutaneous, articular, or renal involvement. The abdominal involvement was significantly (*p* < 0.03) more severe in patients with scrotal or penoscrotal involvement than in subjects with isolated penile involvement.

The clinical diagnosis of Henoch–Schönlein syndrome was supported by 24 extragenital (skin, N = 22; kidney, N = 2) and eight genital (testicle, N = 6; appendix testis, N = 1; appendix epididymis, N = 1) histopathology studies. Immunofluorescence testing for immunoglobulin A deposits was performed in 13 cases (skin, N = 10; kidney, N = 2; testicle, N = 1) and all had positive results.

#### 3.2.2. Penile Involvement

Swelling, erythema, purpuric rash, and pain were the most-reported manifestations in the 37 cases with penile involvement (Table 3). Transient micturition disorders induced by penile swelling were observed in a 2- and an 11-year-old patient [18,94]. Placement of a suprapubic catheter for one week was necessary in the latter case [94].

A priapism was observed in two cases. In a 9-year-old boy with erection of the penis for 9 h, detumescence occurred after caudal anesthesia [15]. In a 37-year-old man with an inherited thrombophilia secondary to a prothrombin gene mutation, a bilateral deep vein thrombosis of the lower extremities and a thrombosis of the dorsal penile vein, causing priapism, were noted. Despite systemic anticoagulation and intracavernous irrigation of epinephrine and streptokinase, an inability to obtain an erection firm enough for sexual intercourse was observed. Consequently, a penile prosthesis was implanted [16].

The duration of penile involvement was documented in 24 of the 37 cases. Seventeen patients were treated with corticosteroids and seven without. The duration of penile involvement after diagnosis was 4 [2,3,4,5,6] days in cases with steroids and 9 [6,7,8,9,10,11,12,13,14,15,16,17] days in cases without (*p* < 0.05).

#### 3.2.3. Scrotal Involvement

**Figure 2:**
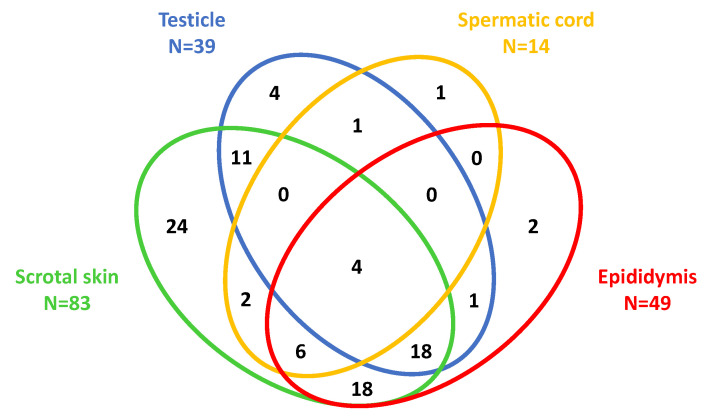
Scrotal skin inflammation, epididymitis, orchitis, and funiculitis in males with Henoch–Schönlein syndrome.

A scrotal involvement occurred in 96 cases. The main presenting features were pain (N = 85; 89%), swelling (N = 79; 82%), erythema (N = 42; 44%), and scrotal purpura (N = 22; 23%). Pain was reported by all patients with scrotal content (i.e., epidydimal, testicular or spermatic cord) involvement. A surgical scrotal exploration was performed in 26 (27%) cases. A total of 213 anatomical scrotal structures were involved in the aforementioned patients (Table 4). The scrotal skin (86%), the epididymis (51%), the testes (41%), and the spermatic cord (15%) were, in descending order of frequency, the most affected structures. Twenty-three cases presented both with epididymitis and orchitis (for this association both the term epididymo-orchitis and “testiculitis” are used). The involvement of the scrotal skin was mostly bilateral; that of the epididymis, testes, and spermatic cord were mostly (*p* < 0.0001) unilateral (with a predilection for the left side). The Venn diagram depicting the cases with scrotal skin, testicular, spermatic cord, and epididymal involvement appears in Figure 2.

An ischemic testicular lesion was noted in nine cases who presented with pain (N = 9), scrotal swelling (N = 7), and erythema (N = 4). The diagnosis of ischemic testicular damage without torsion [32,54,78,79,81] was made in five cases (5, 8, 11, 12 and 24 years of age), and an orchidectomy was performed in four of the five cases [32,54,78,79]. The diagnosis of testicular torsion [27,40,76,83] was made in four cases (2, 4, 5, and 6 years of age); a testicular detorsion was performed in all of them, and was immediately followed by a normal testicular perfusion [27,40,76,83].

The duration of the scrotal involvement after diagnosis was documented in 53 of the 96 cases. Twenty-nine patients were treated with corticosteroids and twenty-four were treated without. The duration of scrotal involvement was 4 [2,3,4,5,6,7,8,9,10] days in cases with steroids and 3 [2,3,4,5,6] days in cases without (*p* = 0.445).

## 4. Discussion

The external genitalia are implicated in every fifth male with Henoch–Schönlein syndrome [1,2]. Integrating and complementing the results of a recent review analyzing Henoch–Schönlein syndrome with scrotal involvement in 21 children [82], the discussion of this review will address the temporal relationship between the distinctive features of this vasculitis and the genital participation, the histopathology features, and the characteristics of penile and scrotal involvement, with emphasis on the ischemic testicular damage, and management.

The scrotal and penile involvement mostly appear concurrently with or after the cutaneous features of Henoch–Schönlein syndrome. However, contrary to a widely held view, genital involvement may occasionally be the presenting clinical sign. Histopathology data demonstrate that the genital disease directly results from the vasculitis process. Local swelling, erythema, and purpuric rash characterize the penile involvement in Henoch–Schönlein syndrome. Micturition disorders and priapism are uncommon complications of penile involvement. Scrotal swelling, erythema (with or without associated purpuric rash), or pain may be induced by a scrotal skin inflammation or, less frequently, by an epididymal or testicular involvement. The right spermatic vein drains directly into the low-pressure inferior caval vein, while on the left side, the spermatic vein joins with the relatively high-pressure renal vein [97]. This anatomic difference likely explains why most cases with testicular, epidydimal, or spermatic chord participation are on the left side or are bilateral. Since vasculitides may result in vessel occlusion, the sporadic occurrence of ischemic testicular damage is not surprising. In contrast, we did not find data in the literature to support the association of Henoch–Schönlein syndrome with testicular torsion.

**Figure 3:**
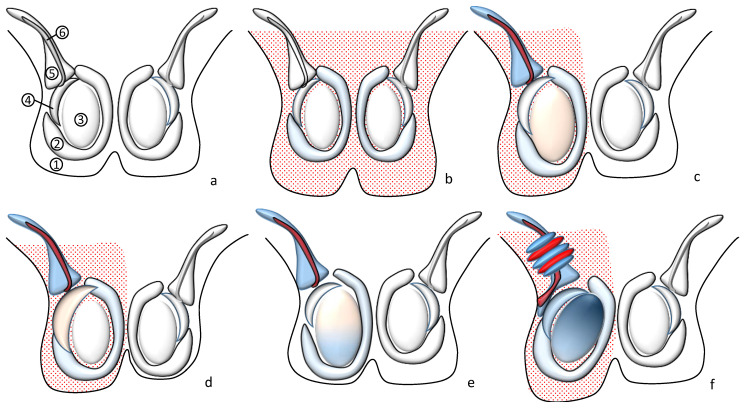
Scrotal involvement in Henoch–Schönlein syndrome. (**a**) Schematic representation of the normal scrotal anatomy: scrotal wall (1), tunica vaginalis (2), testicle (3), epididymis (4), pampiniform plexus (5), spermatic artery (6). (**b**) Scrotal skin inflammation: bilateral thickening and swelling of the scrotal wall, small bilateral hydroceles, normal testes. (**c**) Orchitis: increased testicular volume and blood flow, often thickening of the scrotal wall, hydrocele, and funiculitis. (**d**) Epididymitis: increased epididymal volume and blood flow, often thickening of the scrotal wall, hydrocele, and funiculitis. (**e**) Primary vascular testicular damage: increased testicular volume, focal or diffuse decrease/absence of testicular blood flow, absent twisting of the spermatic cord. (**f**) Testicular torsion: twisting of the spermatic cord, redundant spermatic cord in the scrotal sac, rotated testis, increased volume, decrease in/absence of testicular blood flow, sometimes hydrocele and thickening of the scrotal wall.

Every male Henoch–Schönlein patient with scrotal swelling, erythema, or pain requires careful evaluation [1,2,98,99]. The physical examination includes the inspection of the genital area and the palpation of the scrotal skin along with its content. While the underlying cause in most cases of painless scrotal sac swelling and erythema can be determined by history and physical examination alone, scrotal ultrasound and high-resolution color (and pulsed) Doppler imaging are sometimes crucial [8,9,98,99] to assess and categorize these patients, and particularly to exclude conditions that compromise testicular blood flow (Table 5, Figure 3).

The present data demonstrates that Henoch–Schönlein syndrome is a self-limited condition that usually lasts ≤ 8 weeks in cases both with and without the involvement of external genitalia. Although systemic corticosteroids are often prescribed (and might perhaps reduce the duration of penile involvement), the literature suggests that these agents effectively reduce bothersome symptoms such as abdominal or joint pain, but do not prevent the development of kidney disease [1,2]. Therefore, supportive management is usually advised. Corticosteroids are justified exclusively in cases with a disturbing genital (or, as known, abdominal) involvement.

This review has some limitations. First, it integrates cases published between 1972 and 2022, which were not always well-documented. Second, since clinical practice recommendations can be difficult to infer from the accumulation of case reports, suggested management arises from low-quality evidence. The strength of this review is that it first details the spectrum of conditions that account for an acute involvement of the male external genitalia in Henoch–Schönlein syndrome, including scrotal skin inflammation, epididymitis, orchitis, and ischemic testicular damage (both without and with torsion). Furthermore, we were able to include more than 110 Henoch–Schönlein cases with involvement of the male external genitalia.

In conclusion, Henoch–Schönlein purpura can present with a penile, penoscrotal or scrotal involvement. Scrotal involvement can result from skin inflammation, epididymitis, funiculitis, orchitis, or testicular ischemia, often combined.

## Figures and Tables

**Table 1:** **CAAR** grading for **C**utaneous, **A**bdominal, **A**rticular and **R**enal involvement in Henoch–Schönlein syndrome. The involvement is graded as absent, mild, moderate, or severe [1].

**C**utaneous involvementabsent: - absent: No skin lesions - mild: Skin lesions located on buttocks and lower extremities alone - moderate: Skin lesions located on (a) buttocks **and** lower extremities and (b) either trunk **or** upper extremities - severe: Skin lesions located on (a) buttocks and lower extremities, (b) trunk **and** (c) upper extremities **A**bdominal involvement - absent: No symptoms, no findings - mild: Mild abdominal pain (medically elicited) - moderate: Moderate abdominal pain (transient complaints brought to medical attention) - severe: Severe abdominal pain and/or melena, and/or hematemesis, and/or intussusception **A**rticular involvement - absent: No symptoms, no findings - mild: Symptoms or findings of articular involvement but no functional abnormalities - moderate: Symptoms and findings of articular involvement causing mild functional reduction (e.g., limping) - severe: Symptoms and findings causing moderate functional loss (e.g., inability to walk) **R**enal involvement - absent: Normal urinalysis - mild: Pathological hematuria, normal proteinuria (stick negative or [+]) - moderate: Pathological hematuria, mild-moderate proteinuria (stick + to ++) - severe: Pathological hematuria, severe proteinuria (stick ≥ +++)

**Table 2:** Involvement of male external genitalia in Henoch–Schönlein syndrome. Characteristics of the 114 cases (1.5 to 75 years of age).

	All Cases	Isolated Penile Involvement	Isolated Scrotal Involvement	Penoscrotal Involvement	*p* Value
N	114	18	77	19	
Age					
years	5.8 [4.0–8.0]	4.9 [3.5–7.5]	6.0 [4.0–8.0]	5.0 [4.0–5.8]	0.0586
≤18 years, N	104	16	70	19	0.3600
Precursors					0.5214
Infection	41	9	21	11	
Upper respiratory infection, N	33	6	17	10	
Further infections, N	8	3	4	1	
Vaccine, N	1	0	1	0	
Hymenoptera sting, N	1	0	1	0	
Time relationship					0.2144
Skin before genitalia by ≥3 days *, N	55	10	36	9	
Skin and genitalia concomitant, N	41	4	29	8	
Genitalia before skin by ≥3 days **, N	10	0	9	1	
Information not available, N	8	4	3	1	
**CAAR-grading of organ involvement**					
Cutaneous involvement					0.8483
Mild, N	73	11	49	13	
Moderate, N	26	5	18	3	
Severe, N	11	2	6	3	
Information not available, N	4	0	4	0	
Abdominal involvement					<0.03 ^◆^
None, N	49	13	26	10	
Mild, N	24	5	14	5	
Moderate, N	20	0	17	3	
Severe, N	19	0	18	1	
Information not available, N	2	0	2	0	
Articular involvement					0.1647
None, N	45	5	33	7	
Mild, N	52	10	36	6	
Moderate, N	10	2	3	5	
Severe, N	5	1	3	1	
Information not available, N	2	0	2	0	
Kidney involvement					0.4818
None, N	73	14	48	11	
Mild, N	23	2	16	5	
Moderate, N	14	2	9	3	
Severe, N	2	0	2	0	
Information not available, N	2	0	2	0	

* 3–5 days, N = 16; 6–10 days, N = 16; 11–30 days, N = 17; >30 days, N = 6; ** 3–10 days, N = 9; 11–30 days, N = 0; >30 days, N = 1; ◆ penile versus scrotal or penoscrotal involvement.

**Table 3:** Symptoms and clinical findings of penile involvement in 37 Henoch–Schönlein patients.

Penile Manifestations	N (%)
Swelling, N	26 (70%)
Erythema, N	23 (62%)
Purpuric rash, N	20 (54%)
Pain, N	15 (40%)
Transient micturition disorders, N	2 (5%)
Priapism, N	2 (5%)

**Table 4:** Scrotal structures involved in 96 males with Henoch–Schönlein syndrome.

		Laterality			
	Total N = 213	Left N = 82	Right N = 38	Bilateral N = 83	Not Specified N = 10
**Scrotal skin inflammation, N**	**83**	**22**	**10**	**51**	**0**
**Epididymitis, N**	**49**	**26**	**6**	**12**	**5**
**Testicular involvement, N**	**39**	**15**	**10**	**14**	**0**
Orchitis, N	30	10	7	13	0
Ischemic damage, N	9	5	3	1	0
Without torsion, N	5	3	1	1	0
With torsion, N	4	2	2	0	0
**Spermatic cord involvement, N**	**14**	**7**	**5**	**0**	**2**
Funiculitis, N	13	6	5	0	2
Spermatic vein thrombosis, N	1	1	0	0	0
**Hydatid torsion, N**	**3**	**3**	**0**	**0**	**0**
**Poorly defined intrascrotal inflammation, N**	**13**	**4**	**3**	**3**	**3**
**Hydrocele, N**	**12**	**5**	**4**	**3**	**0**

**Table 5:** Henoch–Schönlein syndrome with scrotal involvement. History, physical examination, imaging studies, and differential diagnosis.

		Imaging Studies		
	History—Examination	Ultrasound	Color Doppler	Differential Diagnosis
Scrotal skin inflammation	Erythema (sometimes with purpuric lesions), warmth and swelling (painless or only mildly painful) of the scrotal sac (usually bilateral)	Thickening and swelling of the scrotal sac, small bilateral hydroceles, normal testes	Hypervascularity of the scrotal sac (fountain sign)	Acute idiopathic scrotal edema
Orchitis (sometimes associated with epididymitis)	Testicular pain, swelling and warmth, often associated with swelling and erythema of the scrotal sac (usually unilateral)	Increased testicular volume with focal or diffuse hypoechogenicity, often mild-moderate thickening of the scrotal wall, hydrocele (anechoic or with debris), funiculitis	Increased testicular (and, often, epidydimal) blood flow	Reperfusion after intermittent torsion
Epididymitis (mostly associated with orchitis)	Pain posterior to the testicle, often associated with swelling and erythema of the scrotal sac (usually bilateral)	Increased epididymal volume with heterogeneous echogenicity, often mild-moderate thickening of the scrotal wall, hydrocele (anechoic or with debris), funiculitis	Increased epidydimal (and, often, testicular) blood flow	Reperfusion after intermittent torsion
Primary vascular testicular damage	Acute or subacute unilateral testicular pain, testicle usually tender and swollen	Increased testicular volume, focal or diffuse hypoechogenicity, absent twisting of the spermatic cord	Focal or diffuse decrease or absence of testicular blood flow	Focal post-traumatic infarction (due to increased pressure resulting in venous obstruction)
Testicular torsion	Abrupt onset of severe unilateral testicular or scrotal pain—testicle usually tender, swollen, and slightly elevated because of shortening of the cord from twisting—often nausea and vomiting	Twisting of the spermatic cord (whirlpool sign), redundant spermatic cord in the scrotal sac, testis rotated with increased volume, heterogeneous hyperechogenicity, sometimes hydrocele (anechoic or with debris) and mild-moderate thickening of the scrotal wall.	Diffuse decrease or absence of testicular blood flow (pulsed Doppler: absence or reduced first venous and then arterial blood flow)	

## Data Availability

The data supporting this study are available from the corresponding author upon reasonable request.
